# A Dose of Clarity: Decoding the Readability of Online Resources on Steroid Knee Injections

**DOI:** 10.1177/23743735251385918

**Published:** 2025-10-17

**Authors:** Samher Jassim, Sinead Cummins, Conor J Kilkenny, Brendan O’Daly

**Affiliations:** Department of Orthopaedics, 57976 Tallaght University Hospital, Dublin, Ireland

**Keywords:** health literacy, health communication, readability analysis, knee osteoarthritis

## Abstract

This study evaluates the readability and quality of online resources on steroid knee injections. Online materials were identified using Google, Bing, and Yahoo with the search terms steroid knee injection, corticosteroid knee injection, and knee injection treatment. Of 150 screened web pages, 57 met inclusion criteria. Quality was assessed using the DISCERN instrument and *Journal of the American Medical Association* (JAMA) benchmark, while readability was measured using the Flesch-Kincaid Grade Level (FKGL) and Simple Measure of Gobbledygook (SMOG). Health On the Net Foundation Code of Conduct certification status was recorded. The mean DISCERN score was 42.47 ± 17.06, and the *Journal of the American Medical Association* score was 1.58 ± 1.52, indicating low quality. Readability analysis showed an FKGL score of 9.19 ± 2.08 and an SMOG score of 8.20 ± 5.23, suggesting most materials require advanced literacy. For-profit web pages had lower quality but were easier to read, whereas nonprofit and academic sites provided higher quality but more complex content. Most web pages offer low-quality, difficult-to-understand information. Patients should seek reliable sources, and oversight is needed to improve quality and accessibility.

## Introduction

Steroid knee injections are widely used to manage knee pain and inflammation associated with conditions such as osteoarthritis, rheumatoid arthritis, and other inflammatory joint disorders.^1^ As the prevalence of these conditions continues to rise, an increasing number of patients are seeking information online about steroid injections and other treatment options.^2^ This shift toward digital health information reflects the growing reliance on the internet as a primary source of medical knowledge for patients. However, while the internet offers easy access to a vast amount of information, studies have shown that the readability of online health resources often exceeds the recommended 6th–8th grade reading level, a standard endorsed by organizations such as the American Medical Association (AMA) and the National Institutes of Health.^3^ This discrepancy between the reading levels of available resources and the needs of the general population can significantly hinder patient comprehension and limit the effectiveness of online health materials.^4^

The information found on the internet may be inaccurate or misleading. As a result, patients relying on incorrect information may experience treatment failures or irreversible damage to their health.^5^ A recent study evaluating health information on the internet reported that 40% of the included web pages contained incorrect information.^6^ Given the increasing reliance on the internet for medical knowledge, it is critical to assess whether the information available is both appropriate and reliable.^7^

The ability of individuals from different socioeconomic and educational backgrounds to acquire and comprehend health information varies significantly. Low health literacy negatively impacts health outcomes across various medical disciplines, including orthopedics. A recent study found that patient education materials on knee osteoarthritis are often written at a 10th–12th grade level rather than the recommended 6th–8th grade level.^8^ Patients frequently turn to internet resources to evaluate treatment options, including steroid knee injections. It is essential for patients to have access to accurate, understandable information to support informed decision-making.^9^

In this study, we evaluate the readability of online resources related to steroid knee injections, utilizing readability tools such as the Flesch-Kincaid Grade Level (FKGL) and the Simple Measure of Gobbledygook (SMOG) index to assess the accessibility of the language.^10^ In addition to readability, the quality and reliability of the information are evaluated using the DISCERN tool and the *Journal of the American Medical Association* (JAMA) benchmark.^11^ By examining both readability and quality, this study seeks to provide valuable insights into the current state of online health information on steroid knee injections, highlighting areas for improvement to better support patient understanding and informed decision-making.^12^

## Materials and Methods

### Data Collection

For this content analysis study, 3 search engines: Google, Yahoo, and Bing—were used to conduct the search —the 3 most popular search engines 13. Two search terms, “steroid knee injection information” and “corticosteroid knee injection,” were employed. For each search engine, the top 30 websites per search term were selected, resulting in 60 websites per engine and a total of 180 websites. The Google search engine was accessed on November 13, 2024. A total of 180 websites were analyzed, with 90 websites corresponding to each search term. To avoid bias from previous search results, browsing history and cookies were cleared before each search. Duplicate websites, those requiring membership registration or subscription, irrelevant content, or unreachable links were excluded from the analysis. Each website was categorized into 1 of 3 source types: profit (websites prioritizing monetization, including government and charity sites), nonprofit (websites focused on supporting a cause rather than generating revenue, including government and charity sites), and journals/books.

### Information Quality

The study employed the DISCERN tool and the JAMA scales to evaluate the quality and reliability of the information. High scores on the JAMA and DISCERN scales, along with the presence of the Health On the Net Foundation Code of Conduct (HONCode) seal, were associated with high-quality scientific content. The DISCERN tool, developed in 1998 by Charnock et al, is widely used to assess the content of websites. It comprises 16 items, each scored from 1 to 5. The first 8 items pertain to general information, while the last 7 items focus on treatment and treatment options. A score of 5 is assigned for a definite “yes” answer, 1 for a definite “no” answer, and scores of 2, 3, or 4 for partial responses. DISCERN scores range from a minimum of 16 to a maximum of 80. These total scores are categorized as follows: excellent (63 or above), good (51–62), fair (39–50), poor (28–38), and very poor (16–27).^14^

The JAMA scale was developed by Silberg et al in 1997 to assess the quality of healthrelated websites. It evaluates 4 key characteristics: authorship (including authors, contributors, and affiliations), references (sources and citations), disclosure statements (ownership, conflicts of interest, and currency), and currency (publication dates and updates). Each parameter is scored as either 0 or 1 point, with the total score ranging from 0 to 4. Higher scores indicate higher content quality, and there is no categorization for scores.^15^ The websites were scored by 2 independent reviewers trained in both DISCERN and JAMA criteria. In cases of disagreement, the authors discussed their perspectives and reached a final decision collectively. Additionally, all websites were assessed for HONCode certification. HONCode aims to identify high-quality online information, and website owners can obtain certification by meeting specific criteria that align with its core principles.^1^

The final 3 markers were used to evaluate the quality of online information. In this study, content scoring close to 80 points on the DISCERN scale and a full 4 points on the JAMA scale for each website was proportionally indicative of high information quality. Websites displaying the HONCode seal were also considered to have high-quality content.

### Readability

Readability refers to the ease with which a reader can understand a text, as measured by systematic formulas.^14^ In addition to assessing information quality, a readability analysis was conducted to determine how the content might be perceived by readers. The FKGL and SMOG formulas were used for this analysis. Readability is defined as a person's ability to comprehend a text. The website https://www.webfx.com/tools/read-able/ was used to measure readability, with FKGL and SMOG serving as the primary indicators. Higher scores on these indicators correspond to lower readability. The formulas used to calculate these indicators are illustrated in Table 1.^16^ Elevated readability scores are often associated with reduced accessibility of the content, potentially limiting user comprehension and engagement.

**Table 1.** Readability Formulas.^13^

Parameters	Formula
FKGL	(11.8 × syllables/words) + (0.39 × words/sentences) – 15.59
SMOG	1.0430 × (σ) × (30/sentences) + 3.1291 σ = number of words ≥3 syllables

Abbreviations: FKGL, Flesch-Kincaid Grade Level; SMOG: Simple Measure of Gobbledygook.

### Statistical Analysis

The statistical program IBM SPSS Statistics for Windows, Version 15 (Released 2006; IBM Corp., Armonk, New York, USA) was used. The values were presented as mean, standard deviation, median, minimum, and maximum. For comparing continuous parameters that were not normally distributed in 2 categories, the Mann–Whitney *U* test was applied. For comparing categorical variables, the chi-square test was used. The interrater reliability for DISCERN and JAMA was assessed, and Cohen's kappa values were calculated.

## Results

**Figure 1.**
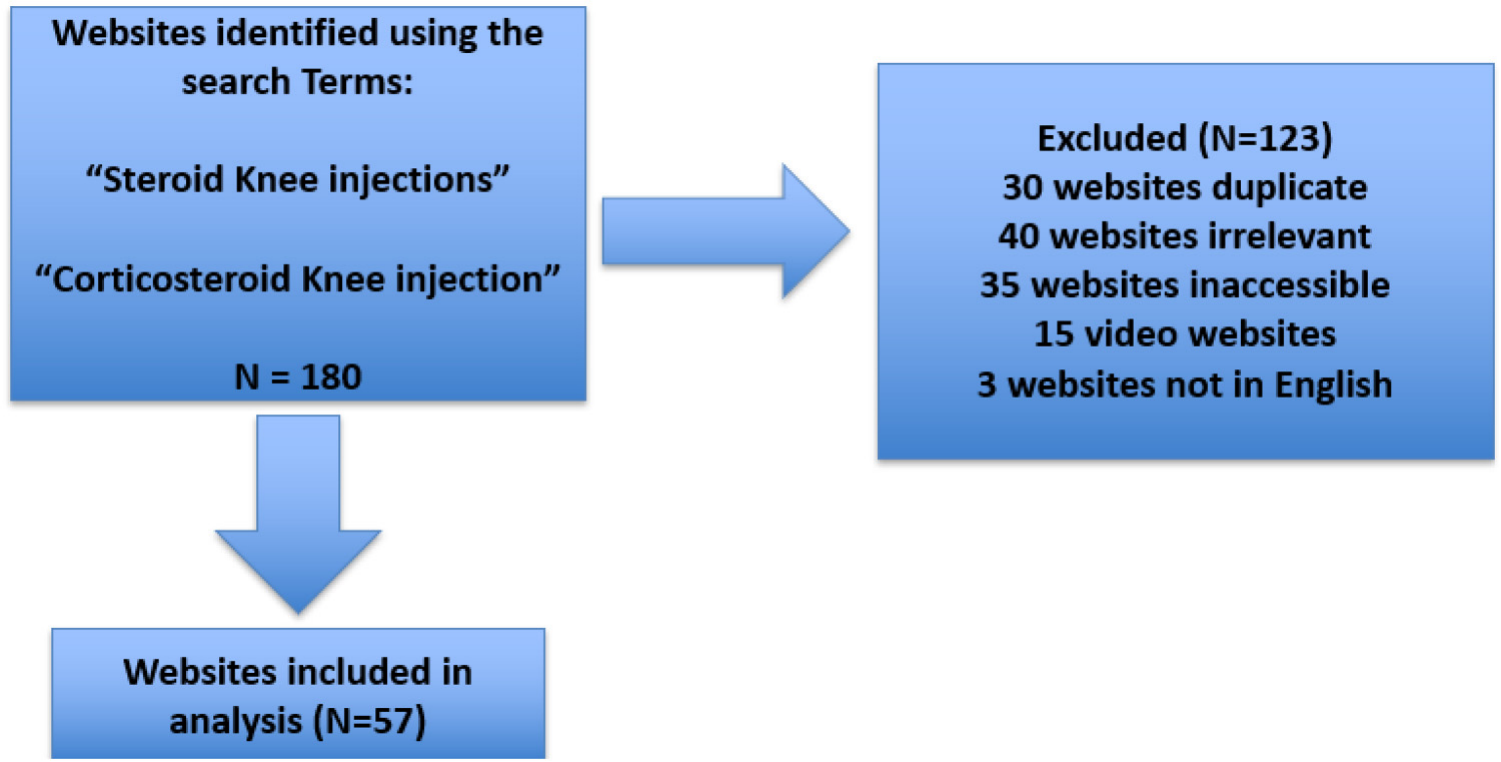
Flowchart of Study.

A total of 180 web pages were initially included, but 40 were excluded due to irrelevance, 35 were inaccessible, 30 were duplicates, 15 were nontext-based content (eg, videos) and 3 were non-English web pages. As a result, 57 web pages were included in the final analysis (Figure 1). The interrater reliability kappa values were calculated as 0.77 for JAMA and 0.72 for DISCERN, indicating substantial agreement between reviewers. The total evaluation scores were found to be 41.41 ± 15.98 for DISCERN and 1.52 ± 1.43 for JAMA. Among the DISCERN subcategories, the highest scores were given to “Is it relevant?” (4.21 ± 0.93) and “Does it achieve its aims?” (3.38 ± 1.14), while the lowest score was given to “Does it describe how the treatment choices affect the overall quality of life?” (2.10 ± 1.16) (Table 2). Of the 57 web pages included in the study, 29 were for-profit, 21 were nonprofit, and 7 were part of the journal–book group. It was observed that 10.52% (*n* = 6) of these web pages had the HONCode seal. This rate was determined to be 42.85% in the journal group, 9.52% in the nonprofit group, and 3.44% in the for-profit group (*p* = .068).

**Table 2.** DISCERN, JAMA, and Readability Indicators’ Grade Level.

DISCERN Questions	Mean ± SD
Overall DISCERN Score	41.41 ± 15.98
1. Are the aims clear?	3.40 ± 1.04
2. Does it achieve its aims?	3.38 ± 1.14
3. Is it relevant?	4.21 ± 0.93
4. Is it clear what sources of information were used (other than the author or producer)?	1.87 ± 1.61
5. Is it clear when the information used or reported in the publication was produced?	2.10 ± 1.60
6. Is it balanced and unbiased?	2.85 ± 1.30
7. Does it provide details of additional sources of support and information?	2.40 ± 1.17
8. Does it refer to areas of uncertainty?	2.50 ± 1.13
9. Does it describe how each treatment works?	2.90 ± 1.17
10. Does it describe the benefits of each treatment?	2.65 ± 1.18
11. Does it describe the risks of each treatment?	2.45 ± 1.17
12. Does it describe what would happen if no treatment is used?	2.15 ± 1.16
13. Does it describe how the treatment choices affect the overall quality of life?	2.10 ± 1.16
14. Is it clear that there may be more than one possible treatment choice?	2.38 ± 1.12
15. Does it provide support for shared decision-making?	2.42 ± 1.08
JAMA	1.52 ± 1.43
FKGL	9.05 ± 1.95
SMOG	7.87 ± 4.7

Abbreviations: JAMA, *Journal of the American Medical Association*; FKGL, Flesch-Kincaid Grade Level; SMOG, Simple Measure of Gobbledygook.

The readability indicators for all web pages were found to be 9.05 ± 1.95 for FKGL and 7.87 ± 4.7 for SMOG. The highest readability scores were observed in the journal–book group, while the lowest were in the for-profit group (*p* = .001 and .002, respectively).

The DISCERN score was found to be x for the journal-book group, x for the nonprofit group, and x for the profit group, and there was a significant difference between all groups based on subgroup analysis (*p* < .001). Similarly, the JAMA score was found to be highest in the journal–book group and lowest in the profit group (*p* < .001). When groups were compared, the readability indicators SMOG and FKGL were found to be highest in the journal–book group and lowest in the profit group. However, the high readability scores of journal–book websites indicate low readability, while the low readability scores of profit websites indicate high readability levels (Table 3).

**Table 3.** Quality and Readability Scores of Website Material According to Sources.

	Profit (*n* = 29)	Nonprofit (*n* = 21)	Journal-book (*n* = 7)	p
DISCERN	30 (17-43)	50 (35-65)	63 (49-77)	<.001
JAMA	0.8 ± 1.51	2.1 ± 1.37	2.8 ± 1.23	<.001
HONCODE, *n* (%)	1 (3.44)	2 (9.52)	3 (42.5)	.068*
FKGL	8.55 ± 2.06	9.45 ± 1.87	9.95 ± 1.68	.001
SMOG	7.10 ± 4.5	8.40 ± 4.8	9.50 ± 5.2	.002

Abbreviations: JAMA, *Journal of the American Medical Association*; FKGL, Flesch-Kincaid Grade Level; SMOG, Simple Measure of Gobbledygook.

## Discussion

The internet has emerged as a key resource for medical information, widely used by both patients and healthcare professionals. Nonetheless, the reliability and quality of online health information remain uncertain, largely due to the absence of regulatory oversight. This variability often makes it challenging for patients to locate precise and dependable information about steroid knee injections.^17^

To the best of our knowledge, this study is the first to assess the quality and readability of online information regarding “Corticosteroid Knee Injections” based on the existing literature. This research, which simultaneously evaluated information quality and readability, found that many for-profit sites provided low-quality information. The average DISCERN score in our study was determined to be 41.41 ± 15.98. The DISCERN tool assesses the quality of health information on web pages by considering various factors, such as clearly referencing information sources, maintaining a balanced and unbiased perspective, comprehensively outlining the benefits and risks of each treatment option, and supporting shared decision-making. While the ideal web page would score 80 points, none of the analyzed sites, including academic ones, achieved this benchmark. Significant variations in DISCERN scores were observed among the web pages related to corticosteroid knee injections. Notably, higher scores were predominantly associated with journal and book-based web pages, whereas lower scores were more commonly linked to for-profit web pages.

Similar studies have reported higher DISCERN scores for journal and book-based web pages, with findings consistent across various medical fields. For instance, a study evaluating online information about rotator cuff repairs found that academically affiliated web pages achieved the highest DISCERN scores (51.6), significantly outperforming private physician and news web pages.^13^ Another study assessing shoulder pain web pages reported an average DISCERN score of 50.92, with a strong bias toward biomedical information and limited focus on psychosocial aspects.^14^ Interestingly, it was noted that even web pages with relatively high DISCERN scores could occasionally include inaccurate or biased information. Factors such as the lack of regulatory oversight, the presence of misleading advertisements, and the influence of personal biases in online health content can significantly impact the quality, reliability, and overall scores of the information provided.

In our study, the average JAMA score was determined to be 1.52 ± 1.43, indicating that, on average, most web pages met fewer than half of the 4 JAMA criteria. Profit-based web pages typically scored between 0 and 2 on the JAMA scale, whereas journal and book-based web pages scored between 3 and 4. This discrepancy raises further concerns about the reliability of information on profit-driven sites, which are often more accessible and frequently accessed by patients.

The HONcode is a certification system developed to ensure that health-related web pages adhere to established ethical standards. Created by the Health On the Net Foundation, based in Switzerland, its purpose is to provide users with reliable, accurate, and current health information on the internet. Web pages that meet the HONcode requirements, such as ensuring quality, transparency, proper citation of sources, privacy protection, and user-friendly design, can earn the “HONcode certified” label. These criteria are intended to promote the dissemination of trustworthy and credible information.^15^ In our study, we found that only 10.52% of the web pages assessed had HONcode certification. This percentage is lower than those reported in similar studies, such as the evaluation of rotator cuff repair web pages (11% HONcode-certified).^1^ These differences highlight the variability in the quality and reliability of online health information across different medical topics.

Our study revealed that profit-based web pages were the most common type, accounting for 29 out of the 57 web pages evaluated. The quality and quality of information on these profit-driven sites were notably low, making it challenging for patients to access correct and reliable information. Similar findings have been reported in other studies.^16^ Profit web pages consistently scored the lowest in HONCode certification, DISCERN, and JAMA evaluations. Interestingly, their readability levels were higher compared to journal–book, and nonprofit web pages, a trend also observed in other studies when reviewing the literature.^18^

The role of healthcare professionals in guiding patients toward trustworthy online resources is crucial. In today's digital era, patients often rely on the internet for health-related information, but the quality and reliability of such information are not always guaranteed. Healthcare professionals can play a vital role by directing patients to credible online sources. This guidance helps patients better understand their health conditions and make informed decisions, particularly for those with limited health literacy who may struggle with medical terminology or treatment plans. By providing clear and accessible information, healthcare professionals can bridge this gap and enhance patient education.

Furthermore, clinicians have a key role in addressing the challenges highlighted by the study's findings, many of which stem from communication barriers between patients and the healthcare system. Clinicians are uniquely positioned to close these gaps and improve the effectiveness of patient care. By promoting the use of reliable digital tools and trustworthy online resources, clinicians can empower patients to take a more active and informed role in managing their health. This approach not only improves access to accurate information but also encourages patients to engage more proactively in their healthcare journey.^19^

In this study, we used the FKGL and SMOG tools to evaluate the readability of web pages. The FKGL score was originally developed in 1975 to assess the readability of military manuals for the US Navy.^18^ A score below 8 is considered ideal for universal accessibility and readability. While FKGL emphasizes sentence length over word length, SMOG focuses exclusively on the presence of multisyllabic words and evaluates text on a lexical rather than syntactical basis. SMOG is valid only for English and is widely used in health information studies, with a score of 10 or lower recommended for universal readability.^20^ In our study, the average FKGL and SMOG scores were 9.05 ± 1.95 and 7.87 ± 4.7, respectively. These results indicate that most of the evaluated web pages are written at a 9th–10th-grade reading level, which may present challenges for patient comprehension. For comparison, a study evaluating rotator cuff repair web pages reported an average FKGL score of 10.98, corresponding to an 11th-grade reading level.^19^ Similarly, Barrett et al^16^ found an average FKGL score equivalent to a 7th- or 8th-grade reading level in their assessment of shoulder pain web pages, highlighting variability in readability across different medical topics.^18^

Interestingly, for-profit web pages tended to have lower FKGL and SMOG scores, indicating higher readability compared to other types of web pages. However, this does not necessarily imply that the information on these sites is reliable or up to date. To enhance readability and ensure better comprehension, the AMA recommends that patient education materials be written at a 7th-grade reading level or lower.^2^

## Limitations

This study has several limitations. While the readability assessment tools FKGL and SMOG are objective and reproducible for text-based content, they do not account for audio, visual, or video-based information, a limitation noted in other similar studies.^20^ Additionally, the study relied on a single search engine, and the dynamic nature of search engine algorithms means that results can vary over time and across different geographic locations. Another potential limitation is the ever-changing nature of web content, as information on web pages can evolve or be updated after the evaluation period. Lastly, although the DISCERN and JAMA criteria are objective measures, the evaluators’ interpretations may introduce subjectivity into the assessment process.

## Conclusions

In conclusion, the quality and readability metrics used in this study on steroid knee injections align with findings from similar studies on other medical topics. A significant portion of the web pages analyzed exhibited financial bias and provided low-quality health information. Many patients may struggle to access reliable and comprehensible online resources regarding steroid knee injections. To improve the accessibility and reliability of online health information, it is essential that patients seek information from reputable sources such as government health web pages, academic institutions, and peer-reviewed articles. There is a need for mechanisms to systematically monitor and improve the quality and readability of online medical information. Ensuring the quality and accessibility of health content is crucial for enhancing public health and promoting informed decision-making.

## References

[1] McAlindon TE Bannuru RR Sullivan MC , et al. OARSI guidelines for the non-surgical management of knee osteoarthritis. Osteoarthritis Cartilage. 2014;22(3):363–88. doi:10.1016/j.joca.2014.01.003 24462672

[2] Fox S . Information triage. Pew Research Center. Published April 14, 2024. https://www.pewresearch.org/internet/2013/01/15/information-triage/

[3] Mcinnes N Haglund BJA . Readability of online health information: implications for health literacy. Inf Health Social Care. 2011;36(4):173–89. doi:10.3109/17538157.2010.542529 21332302

[4] Berkman ND Sheridan SL Donahue KE Halpern DJ Crotty K . Low health literacy and health outcomes: an updated systematic review. Ann Intern Med. 2011;155(2):97–107. doi:10.7326/0003-4819-155-2-201107190-00005 21768583

[5] Tan SSL Goonawardene N . Internet health information seeking and the patient–physician relationship: a systematic review. J Med Internet Res. 2017;19(1):e9. doi:10.2196/jmir.5729 PMC5290294 28104579

[6] Cline RJW . Consumer health information seeking on the internet: the state of the art. Health Educ Res. 2001;16(6):671–92. doi:10.1093/her/16.6.671 11780707

[7] Eysenbach G Powell J Kuss O Sa ER . Empirical studies assessing the quality of health information for consumers on the world wide web. JAMA. 2002;287(20):2691. doi:10.1001/jama.287.20.2691 12020305

[8] Kincaid JP, Fishburne RP Jr, Rogers RL, et al. *Derivation of new readability formulas (automated readability index, fog count and Flesch reading ease formula) for Navy enlisted personnel*. Institute for Simulation and Training, Research Report. ERIC Document ED108134, 1975.

[9] Zhang B . Analysis of text readability of college English course books. OALib. 2021;08(09):1–14. doi:10.4236/oalib.1107817

[10] Charnock D Shepperd S Needham G Gann R . DISCERN: an instrument for judging the quality of written consumer health information on treatment choices. J Epidemiol Community Health. 1999;53(2):105–11. doi:10.1136/jech.53.2.105 10396471 PMC1756830

[11] Silberg WM . Assessing, controlling, and assuring the quality of medical information on the internet. JAMA. 1997;277(15):1244. doi:10.1001/jama.1997.03540390074039 9103351

[12] Shimizu T Kimura K Sugihara E , et al. MEK inhibition preferentially suppresses anchorage-independent growth in osteosarcoma cells and decreases tumors in vivo. J Orthop Res. 2021;39(12):2732–43. doi:10.1002/jor.25023 33751653

[13] Daraz L Morrow AS Ponce OJ , et al. Readability of online health information: a meta-narrative systematic review. Am J Med Qual. 2018;33(5):487–92. doi:10.1177/1062860617751639 29345143

[14] Buford DA . Restoration of the rotator cuff footprint after arthroscopic single row repair (SS-01). Arthroscopy J Arthroscopic Relat Surg. 2010;26(6):e1. doi:10.1016/j.arthro.2010.04.011

[15] CÉLia NB Vincent NB Antoine NG . Evolution of health web certification through the HONcode experience. Stud Health Technol Inf. 2011;169:53–7. doi:10.3233/978-1-60750-806-9-53 21893713

[16] Barrett DR Boone JD Butch JO Cavender JA Sole G Wassinger CA . A critical appraisal of web-based information on shoulder pain comparing biomedical vs. psychosocial information. J Shoulder Elbow Surg. 2022;32(1):e23–32. doi:10.1016/j.jse.2022.07.023 36108880

[17] Musa M Zeppieri M Atuanya GN , et al. Nutritional factors: benefits in glaucoma and ophthalmologic pathologies. Life. 2023;13(5):1120. doi:10.3390/life13051120 37240765 PMC10222847

[18] Kelly NEW Murray KE McCarthy C O’Shea DB . An objective analysis of quality and readability of online information on COVID-19. Health Technol (Berl). 2021;11(5):1093–9. doi:10.1007/s12553-021-00574-2 34189011 PMC8222704

[19] Warren E Hurley ET Park CN , et al. Evaluation of information from artificial intelligence on rotator cuff repair surgery. JSES International. 2023;8(1):53–7. doi:10.1016/j.jseint.2023.09.009 38312282 PMC10837709

[20] Sabharwal S Badarudeen S Kunju SU . Readability of online patient education materials from the AAOS web site. Clin Orthop Relat Res. 2008;466(5):1245–50. doi:10.1007/s11999-008-0193-8 18324452 PMC2311488

